# Supplementary material for: Mechanism of 2′-fucosyllactose degradation by human-associated Akkermansia
Source: J Bacteriol. 2024 Feb 1;206(2):e00334-23. doi: 10.1128/jb.00334-23 (PMC10886448; doi:10.1128/jb.00334-23)
Supplement: File S2 — R markdown file of basic code and outputs produced during analysis of A. biwaensis CSUN-19. [file jb.00334-23-s0002.html]

 Mechanism of 2’-Fucosyllactose degradation by Human-Associated Akkermansia 


Code 

- Show All Code
- Hide All Code

# Mechanism of 2’-Fucosyllactose degradation by Human-Associated *Akkermansia*

#### *2023-10-13*

## 0.1 Introduction

Among the first bacteria to colonize the human gut of breastfed
infants are bacteria capable of fermenting human milk oligosaccharides
(HMOs). One of the most abundant HMOs, 2’-fucosyllactose (2’-FL), may
specifically drive colonization of the intestine. Recently, differential
growth has been observed across multiple species of *Akkermansia*
on various HMOs including 2’FL. However, the mechanisms of
*Akkermansia* growth on HMOs is poorly understood. In culture, we
found growth of two species, *A. muciniphila* MucT and *A.
biwaensis* CSUN-19, corresponded to a decrease of 2’-FL and an
increase in lactose, indicating that the first step in 2’-FL catabolism
is the cleavage of fucose. Using phylogenetic analysis and
transcriptional profiling, we found that each strain has differences in
the number and expression of fucosidase genes from two glycoside
hydrolase (GH) families: GH29 and GH95. Analysis of gene expression
profiles during mid-log phase growth of both strains revealed that
several GH29 genes were upregulated on 2’-FL, whereas the GH95 genes
were upregulated only in *A. muciniphila* MucT. Subsequent
cloning and activity assays of one putative fucosidase and
𝛽-galactosidase from *A. biwaensis* CSUN-19 demonstrated these
enzymes are involved in 2’-FL breakdown. Results from this study show
that diverse strains of human associated *Akkermansia* have
machinery to degrade and grow using 2’-FL. Further, these HMO degrading
abilities demonstrate the potential for *Akkermansia* to
influence the development of the gut microbiome early in life and
expands the known metabolic capabilities of this important human
symbiont.

This reproducible and dynamic report was created using Rmarkdown and
the Knitr package, and
summarizes the basic code and outputs (plots, tables, etc) produced
during the course. To analyze RNA Seq data, scripts written by the DIY Transcriptomics course
were used with some exceptions (*EnhancedVolcano*).

---

## 0.2 R packages used

A variety of R packages was used for this analysis. All graphics and
data wrangling were handled using the tidyverse suite of packages. All
packages used are available from the Comprehensive R Archive Network
(CRAN), Bioconductor.org, or Github.

```
library(cowplot)
library(dplyr)
library(DT)
library(edgeR)
library(EnhancedVolcano)
library(gplots) #the heatmap2 function --> heatmaps
library(ggplot2)
library(ggrepel)
library(gt)
library(limma) # venerable package for differential gene expression using linear modeling
library(matrixStats)
library(plotly)
library(RColorBrewer) #need colors to make heatmaps
library(rhdf5)
library(tidyverse)
library(tximport)
```

---

## 0.3 Read mapping

### 0.3.1 Aligning raw reads with Kallisto

Raw reads were mapped to the *Akkermansia biwaensis* CSUN-19
reference transcriptome using Kallisto, version
0.46.2.

---

### 0.3.2 Importing count data into R

After read mapping with Kallisto, TxImport
was used to read Kallisto outputs into the R environment. Results of
Kallisto are abundance measurements (abundance.tsv) as estimated counts
and transcripts per million (TPM). There are 7 abundance measurements
for *A. biwaensis* CSUN-19 (CSUN\_191, CSUN\_192, CSUN\_193,
CSUN\_194, CSUN\_195, CSUN\_196, CSUN\_197).

```
library(tidyverse)
library(tximport)

targets <- read_tsv("CSUN19StudyDesign.txt") # Contains the two experimental sugars Glc and 2FL and the corresponding samples, read in your study design
path <- file.path(targets$sample, "abundance.tsv") # set file paths to your mapped data
Tx = read.table(file='CSUN_19_Tx_file.txt', header=TRUE,sep="") # Reading in the CSUN-19 reference genome
Tx <- as_tibble(Tx)
Tx <- dplyr::select(Tx, "target_id", "gene_name")
Txi_gene_CSUN19 <- tximport(path, 
                          type = "kallisto", 
                          tx2gene = Tx, 
                          txOut = FALSE, 
                          countsFromAbundance = "lengthScaledTPM",
                          ignoreTxVersion = FALSE, ignoreAfterBar = FALSE)
```

---

## 0.4 Preprocessing

### 0.4.1 Impact of filtering and normalization

```
library(tidyverse)
library(edgeR)
library(matrixStats)
library(cowplot)
library(dplyr)

myTPM <- Txi_gene_CSUN19$abundance # abundance data are in TPM, while the counts are read counts mapping to each gene
myCounts <- Txi_gene_CSUN19$counts
targets # a simple table with samples and treatment (Glc and 2FL)
```

```
sampleLabels <- targets$sample

myDGEList <- DGEList(myCounts)
cpm <- cpm(myDGEList) # counts per million of DGEList
CSUN19.log2.cpm <- cpm(myDGEList, log=TRUE) 
CSUN19.log2.cpm.df <- as_tibble(CSUN19.log2.cpm, rownames = "geneID")
CSUN19.log2.cpm.df # DGEList counts are in cpm and log2 converted, unfiltered, and non-normalized
```

```
# add your sample names to this dataframe (we lost these when we read our data in with tximport)
colnames(CSUN19.log2.cpm.df) <- c("geneID", sampleLabels)
# use the tidy package to 'pivot' your dataframe (from wide to long)
CSUN19.log2.cpm.df.pivot <- pivot_longer(CSUN19.log2.cpm.df, # dataframe to be pivoted
                                  cols = CSUN_191:CSUN_197, # column names to be stored as a SINGLE variable
                                  names_to = "samples", # name of that new variable (column)
                                  values_to = "expression") # name of new variable (column) storing all the values (data)

CSUN19.log2.cpm.df.pivot
```

```
# use ggplot
p1 <- ggplot(CSUN19.log2.cpm.df.pivot) +
  aes(x=samples, y=expression, fill=samples) +
  geom_violin(trim = FALSE, show.legend = FALSE) +
  stat_summary(fun = "median", 
               geom = "point", 
               shape = 95, 
               size = 10, 
               color = "black", 
               show.legend = FALSE) +
  labs(y="log2 expression", x = "sample",
       title="Log2 Counts per Million (CPM)",
       subtitle="unfiltered, non-normalized",
       caption=paste0("produced on ", Sys.time())) +
  theme_bw()

# Filtering
keepers <- rowSums(cpm>1)>=3 # filter out cpm with less than 1 count in at least 3 samples or more
CSUN19.myDGEList.filtered <- myDGEList[keepers,] # create DGEList with filtered values
dim(CSUN19.myDGEList.filtered) # look at dimensions of filtered DGEList
```

```
## [1] 2557    7
```

```
CSUN19.log2.cpm.filtered <- cpm(CSUN19.myDGEList.filtered, log=TRUE)
CSUN19.log2.cpm.filtered.df <- as_tibble(CSUN19.log2.cpm.filtered, rownames = "geneID")
colnames(CSUN19.log2.cpm.filtered.df) <- c("geneID", sampleLabels) # attach columns names

CSUN19.log2.cpm.filtered.df # cpm, log2 converted and filtered, non-normalized
```

```
CSUN19.log2.cpm.filtered.df.pivot <- pivot_longer(CSUN19.log2.cpm.filtered.df, # dataframe to be pivoted
                                           cols = CSUN_191:CSUN_197, # column names to be stored as a SINGLE variable
                                           names_to = "samples", # name of that new variable (column)
                                           values_to = "expression") # name of new variable (column) storing all the values (data)
# use ggplot
p2 <- ggplot(CSUN19.log2.cpm.filtered.df.pivot) +
  aes(x=samples, y=expression, fill=samples) +
  geom_violin(trim = FALSE, show.legend = FALSE) +
  stat_summary(fun = "median", 
               geom = "point", 
               shape = 95, 
               size = 10, 
               color = "black", 
               show.legend = FALSE) +
  labs(y="log2 expression", x = "sample",
       title="Log2 Counts per Million (CPM)",
       subtitle="filtered, non-normalized",
       caption=paste0("produced on ", Sys.time())) +
  theme_bw()

# Normalize
myDGEList.filtered.norm <- calcNormFactors(CSUN19.myDGEList.filtered, method = "TMM") # normalize the filtered data with Trimmed Mean of M-Values (TMM)
# use the 'cpm' function from EdgeR to get counts per million from your normalized data
log2.cpm.filtered.norm <- cpm(myDGEList.filtered.norm, log=TRUE)
log2.cpm.filtered.norm.df <- as_tibble(log2.cpm.filtered.norm, rownames = "geneID")
colnames(log2.cpm.filtered.norm.df) <- c("geneID", sampleLabels)

log2.cpm.filtered.norm.df # cpm, log2 converted, filtered and normalized
```

```
# pivot this NORMALIZED data, just as you did earlier
log2.cpm.filtered.norm.df.pivot <- pivot_longer(log2.cpm.filtered.norm.df, # dataframe to be pivoted
                                                cols = CSUN_191:CSUN_197, # column names to be stored as a SINGLE variable
                                                names_to = "samples", # name of that new variable (column)
                                                values_to = "expression") # name of new variable (column) storing all the values (data)

# use ggplot
p3 <- ggplot(log2.cpm.filtered.norm.df.pivot) +
  aes(x=samples, y=expression, fill=samples) +
  geom_violin(trim = FALSE, show.legend = FALSE) +
  stat_summary(fun = "median", 
               geom = "point", 
               shape = 95, 
               size = 10, 
               color = "black", 
               show.legend = FALSE) +
  labs(y="log2 expression", x = "sample",
       title="Log2 Counts per Million (CPM)",
       subtitle="filtered, TMM normalized",
       caption=paste0("produced on ", Sys.time())) +
  theme_bw()

plot_grid(p1, p2, p3, labels = c('A', 'B', 'C'), label_size = 12)
```

Filtering was carried out to remove lowly expressed genes. Genes with
less than 1 count per million (CPM) in at least 3 or more samples
filtered out. This reduced the number of genes from 2587 to 2557.

---

### 0.4.2 Table of filtered and normalized data

```
library(tidyverse)
library(DT) # for making interactive tables
library(plotly) # for making interactive plots
library(gt) # A layered 'grammar of tables' - think ggplot, but for tables

CSUN19.mydata.df <- log2.cpm.filtered.norm.df %>% 
  mutate(glucose.AVG = (CSUN_191 + CSUN_192 + CSUN_193)/3,
         twoFL.AVG = (CSUN_194 + CSUN_195 + CSUN_196 + CSUN_197)/4,
         #now make columns comparing each of the averages above that you're interested in
         LogFC = (twoFL.AVG - glucose.AVG)) %>% 
  mutate_if(is.numeric, round, 2)

CSUN19.mydata.df
```

The table shown below includes expression data for 2557 genes. You
can sort and search the data directly from the table.

---

## 0.5 PCA plot

```
targets
```

```
group <- targets$treatment
group <- factor(group)

pca.res <- prcomp(t(log2.cpm.filtered.norm), scale.=F, retx=T)
pc.var<-pca.res$sdev^2 # sdev^2 captures these eigenvalues from the PCA result
pc.per<-round(pc.var/sum(pc.var)*100, 1) # we can then use these eigenvalues to calculate the percentage variance explained by each PC
pca.res.df <- as_tibble(pca.res$x)

ggplot(pca.res.df) +
  aes(x=PC1, y=PC2, label=sampleLabels, color=group) +
  geom_point(size=4) +
  xlab(paste0("PC1 (",pc.per[1],"%",")")) + 
  ylab(paste0("PC2 (",pc.per[2],"%",")")) +
  labs(title="PCA plot",
       caption=paste0("produced on ", Sys.time())) +
  coord_fixed() +
  theme_bw()
```

---

## 0.6 Volcano plot

```
library(tidyverse)
library(limma) # venerable package for differential gene expression using linear modeling
library(edgeR)
library(gt) 
library(DT) 
library(plotly) 
library(EnhancedVolcano)

# DIFFERENTIAL GENE EXPRESSION ANALYSIS

group <- factor(targets$treatment)
design <- model.matrix(~0 + group)
colnames(design) <- levels(group)
# Use VOOM function from Limma package to model the mean-variance relationship
v.CSUN19.DEGList.filtered.norm <- voom(myDGEList.filtered.norm, design, plot = TRUE)
```

```
# fit a linear model to your data
CSUN19.linearmodelfit <- lmFit(v.CSUN19.DEGList.filtered.norm, design)
CSUN19.contrast.matrix <- makeContrasts(Sugar = TwoFL - Glucose,
                                 levels=design)
CSUN19.linearmodelfits <- contrasts.fit(CSUN19.linearmodelfit, CSUN19.contrast.matrix)
#get bayesian stats for your linear model fit
ebFit <- eBayes(CSUN19.linearmodelfits)
CSUN19.myTopHits <- topTable(ebFit, adjust ="BH", coef=1, number=40000, sort.by="logFC")
# convert to a tibble
CSUN19.myTopHits.df <- CSUN19.myTopHits %>%
  as_tibble(rownames = "geneID")

allgenes <- c("locus_tag=HHJ01_07405", "locus_tag=HHJ01_08010", "locus_tag=HHJ01_10880", "locus_tag=HHJ01_12765", "locus_tag=HHJ01_11495","locus_tag=HHJ01_06070", "locus_tag=HHJ01_04585", "locus_tag=HHJ01_07850") # vector created to label GH29 and GH95 fucosidases in volcano plot

CSUN19Vplot <- EnhancedVolcano(CSUN19.myTopHits.df,
                lab = CSUN19.myTopHits.df$geneID,
                selectLab = allgenes,
                labFace = 'bold', 
                boxedLabels = TRUE,
                labSize = 6,
                typeConnectors = "closed",
                drawConnectors = TRUE,
                widthConnectors = 0.75,
                colConnectors = 'black',
                lengthConnectors = unit(0.01, "npc"),
                maxoverlapsConnectors = 8,
                directionConnectors = 'both',
                x = 'logFC',
                y = 'adj.P.Val',
                xlim = c(-6,6),
                ylim = c(0, 13),
                axisLabSize = 18,
                FCcutoff = 2,
                pCutoff = 0.05,
                cutoffLineType = 'longdash',
                colAlpha = 1,
                cutoffLineWidth = 1,
                legendPosition = 'bottom',
                legendLabSize = 14,
                legendIconSize = 4.0,
                gridlines.major = FALSE,
                gridlines.minor = FALSE,
                border = 'full',
                borderWidth = 0.5,
                raster = FALSE)

#CSUN19Vplot
```

---

## 0.7 Table of DEGs

To identify differentially expressed genes, precision weights were
first applied to each gene based on its mean-variance relationship using
VOOM,
then data was normalized using the TMM
method in EdgeR.
Linear modeling and bayesian stats were employed via Limma
to find genes that were up- or down-regulated in 2FL versus Glc by
4-fold or more, with a false-discovery rate (FDR) of 0.01.

```
results <- decideTests(ebFit, method="global", adjust.method="BH", p.value=0.01, lfc=2)
colnames(v.CSUN19.DEGList.filtered.norm$E) <- sampleLabels
CSUN19.diffGenes <- v.CSUN19.DEGList.filtered.norm$E[results[,1] !=0,]
CSUN19.diffGenes.df <- as_tibble(CSUN19.diffGenes, rownames = "geneID")

CSUN19.diffGenes.df
```

---

## 0.8 Heatmaps and modules

Pearson correlation was used to cluster **527**
differentially expressed genes, which were then represented as heatmap
with the data scaled by Zscore for each row.

```
library(tidyverse)
library(gplots)
library(RColorBrewer)

myheatcolors1 <- bluered(75)
clustRows <- hclust(as.dist(1-cor(t(CSUN19.diffGenes), method="pearson")), method="complete") # cluster rows (genes) by pearson method
clustColumns <- hclust(as.dist(1-cor(CSUN19.diffGenes, method="spearman")), method="complete") #cluster columns by spearman correlation
module.assign <- cutree(clustRows, k=2)
#now assign a color to each module (makes it easy to identify and manipulate)
module.color <- rainbow(length(unique(module.assign)), start=0.1, end=0.9) 
module.color <- module.color[as.vector(module.assign)] 

heatmap.2(CSUN19.diffGenes, 
          Rowv=as.dendrogram(clustRows), 
          Colv=as.dendrogram(clustColumns),
          RowSideColors=module.color,
          col=rev(myheatcolors1), scale='row', labRow=NA,
          density.info="none", trace="none",  
          cexRow=1, cexCol=1, margins=c(8,20), keysize=1.2)
```

## 0.9 Generation of Tables

Data from fucosidases (GH29, GH95) and beta-galactosidases (GH2) of
interest were extracted from general data set using a subsetting
function.

```
library(gt)

# table that has fucosidases as genes of interest (GH29 and GH95)
CSUN19genes <- CSUN19.myTopHits.df[c(251,263,464,764,508,1638,255,1126,2007),c(-4,-5,-7)]

# creates a vector of numbers/characters of the column you are adding
# Order of genes: HHJ01_10880 HHJ01_11495 HHJ01_12765 HHJ01_08010 HHJ01_07405 HHJ01_06700 HHJ01_07850 HHJ01_04585 HHJ01_06070
Annotation <- c('GH29','GH29','GH29','GH29','GH29','GH29','GH95','GH95','GH95')
Clade <- c('Clade 4', 'Clade 7','Clade 5', 'Clade 2', 'Clade 1', 'Clade 3','Clade 2','Clade 1','Clade 3')
# rename the data frame where you will add new column
CSUN19_FUC <-CSUN19genes
# add new column and attach vector you made earlier
CSUN19_FUC$Annotation <- Annotation
CSUN19_FUC$Clade <- Clade
CSUN19_FUCgt <- CSUN19_FUC %>%
  gt() %>%
  cols_move_to_start(columns = c(Annotation, Clade)) %>%
  fmt_number(c('logFC','AveExpr'), decimals = 2) %>%
  fmt_scientific(c('adj.P.Val'), decimals = 2) %>%
  tab_style(locations = cells_column_labels(columns = everything()),
    style = list(cell_borders(sides = "bottom", weight = px(2)),
      cell_borders(sides = "bottom", weight = px(2)),
      cell_text(weight = "bold"))) %>%
  tab_options(column_labels.border.top.color = "white",
              table.border.top.color = "white",
              table.align = "center",
              table.font.names = "arial")
CSUN19_FUCgt
```

| Annotation | Clade | geneID | logFC | AveExpr | adj.P.Val |
| --- | --- | --- | --- | --- | --- |
| GH29 | Clade 4 | locus\_tag=HHJ01\_10880 | 2.59 | 5.25 | 7.08 × 10^−10^ |
| GH29 | Clade 7 | locus\_tag=HHJ01\_11495 | −2.55 | 6.55 | 4.25 × 10^−11^ |
| GH29 | Clade 5 | locus\_tag=HHJ01\_12765 | 2.12 | 5.66 | 1.19 × 10^−7^ |
| GH29 | Clade 2 | locus\_tag=HHJ01\_08010 | 1.64 | 6.48 | 2.42 × 10^−9^ |
| GH29 | Clade 1 | locus\_tag=HHJ01\_07405 | 2.02 | 5.20 | 1.33 × 10^−7^ |
| GH29 | Clade 3 | locus\_tag=HHJ01\_06700 | −0.75 | 8.68 | 1.81 × 10^−6^ |
| GH95 | Clade 2 | locus\_tag=HHJ01\_07850 | −2.58 | 5.04 | 6.08 × 10^−10^ |
| GH95 | Clade 1 | locus\_tag=HHJ01\_04585 | −1.26 | 8.76 | 5.24 × 10^−8^ |
| GH95 | Clade 3 | locus\_tag=HHJ01\_06070 | −0.44 | 7.08 | 1.74 × 10^−3^ |

```
# table of beta-galactosidase gene of interest (GH2)
# table that has genes of interest
CSUN19_GH2 <- CSUN19.myTopHits.df[c(2273,1996,1763,1761,807,1837,1783),c(-4,-5,-7)]
# creates a vector of numbers/characters of the column you are adding
GH2_Annotation <- c('GH2')
# rename the data frame where you will add new column
CSUN19GH2table <- CSUN19_GH2
# add new column and attach vector you made earlier
CSUN19GH2table$Annotation <- GH2_Annotation

CSUN19_GH2gt <- CSUN19GH2table %>%
  gt() %>%
  cols_move_to_start(columns = c(Annotation)) %>%
  fmt_number(c('logFC','AveExpr'), decimals = 2) %>%
  fmt_scientific(c('adj.P.Val'), decimals = 2) %>%
  tab_style(locations = cells_column_labels(columns = everything()),
            style = list(cell_borders(sides = "bottom", weight = px(2)),
                         cell_borders(sides = "bottom", weight = px(2)),
                         cell_text(weight = "bold"))) %>%
  tab_options(column_labels.border.top.color = "white",
              table.border.top.color = "white",
              table.align = "center",
              table.font.names = "arial")
CSUN19_GH2gt
```

| Annotation | geneID | logFC | AveExpr | adj.P.Val |
| --- | --- | --- | --- | --- |
| GH2 | locus\_tag=HHJ01\_05860 | 0.23 | 11.61 | 6.09 × 10^−2^ |
| GH2 | locus\_tag=HHJ01\_10865 | −0.45 | 9.88 | 2.23 × 10^−4^ |
| GH2 | locus\_tag=HHJ01\_11100 | −0.65 | 7.57 | 2.50 × 10^−5^ |
| GH2 | locus\_tag=HHJ01\_11105 | −0.65 | 8.07 | 2.19 × 10^−5^ |
| GH2 | locus\_tag=HHJ01\_11110 | 1.60 | 5.89 | 4.54 × 10^−7^ |
| GH2 | locus\_tag=HHJ01\_11790 | −0.59 | 8.91 | 1.67 × 10^−5^ |
| GH2 | locus\_tag=HHJ01\_03725 | 0.63 | 9.23 | 1.82 × 10^−5^ |

---

## 0.10 Conclusions

Transcriptomic analysis identified genes that may be responsible for
the deconstruction of 2-fucosyllactose (2FL), a sugar found in human
breast milk.

## 0.11 Session info

The output from running ‘sessionInfo’ is shown below and details all
packages and version necessary to reproduce the results in this
report.

```
sessionInfo()
```

```
## R version 4.3.1 (2023-06-16)
## Platform: aarch64-apple-darwin20 (64-bit)
## Running under: macOS Ventura 13.5.1
## 
## Matrix products: default
## BLAS:   /Library/Frameworks/R.framework/Versions/4.3-arm64/Resources/lib/libRblas.0.dylib 
## LAPACK: /Library/Frameworks/R.framework/Versions/4.3-arm64/Resources/lib/libRlapack.dylib;  LAPACK version 3.11.0
## 
## locale:
## [1] en_US.UTF-8/en_US.UTF-8/en_US.UTF-8/C/en_US.UTF-8/en_US.UTF-8
## 
## time zone: America/Los_Angeles
## tzcode source: internal
## 
## attached base packages:
## [1] stats     graphics  grDevices utils     datasets  methods   base     
## 
## other attached packages:
##  [1] tximport_1.28.0        lubridate_1.9.2        forcats_1.0.0         
##  [4] stringr_1.5.0          purrr_1.0.2            readr_2.1.4           
##  [7] tidyr_1.3.0            tibble_3.2.1           tidyverse_2.0.0       
## [10] rhdf5_2.44.0           RColorBrewer_1.1-3     plotly_4.10.2         
## [13] matrixStats_1.0.0      gt_0.9.0               gplots_3.1.3          
## [16] EnhancedVolcano_1.18.0 ggrepel_0.9.3          ggplot2_3.4.3         
## [19] edgeR_3.42.4           limma_3.56.2           DT_0.28               
## [22] dplyr_1.1.2            cowplot_1.1.1          knitr_1.43            
## [25] tinytex_0.46           rmarkdown_2.24        
## 
## loaded via a namespace (and not attached):
##  [1] gtable_0.3.3        xfun_0.40           bslib_0.5.1        
##  [4] htmlwidgets_1.6.2   caTools_1.18.2      lattice_0.21-8     
##  [7] tzdb_0.4.0          rhdf5filters_1.12.1 vctrs_0.6.3        
## [10] tools_4.3.1         bitops_1.0-7        generics_0.1.3     
## [13] parallel_4.3.1      fansi_1.0.4         highr_0.10         
## [16] pkgconfig_2.0.3     KernSmooth_2.23-22  data.table_1.14.8  
## [19] lifecycle_1.0.3     farver_2.1.1        compiler_4.3.1     
## [22] munsell_0.5.0       htmltools_0.5.6     sass_0.4.7         
## [25] yaml_2.3.7          lazyeval_0.2.2      crayon_1.5.2       
## [28] pillar_1.9.0        jquerylib_0.1.4     cachem_1.0.8       
## [31] gtools_3.9.4        tidyselect_1.2.0    locfit_1.5-9.8     
## [34] digest_0.6.33       stringi_1.7.12      labeling_0.4.2     
## [37] fastmap_1.1.1       grid_4.3.1          colorspace_2.1-0   
## [40] cli_3.6.1           magrittr_2.0.3      utf8_1.2.3         
## [43] withr_2.5.0         scales_1.2.1        bit64_4.0.5        
## [46] timechange_0.2.0    httr_1.4.7          bit_4.0.5          
## [49] hms_1.1.3           evaluate_0.21       viridisLite_0.4.2  
## [52] rlang_1.1.1         Rcpp_1.0.11         glue_1.6.2         
## [55] xml2_1.3.5          vroom_1.6.3         rstudioapi_0.15.0  
## [58] jsonlite_1.8.7      R6_2.5.1            Rhdf5lib_1.22.0
```
