# Supplementary material for: Mechanism of 2′-fucosyllactose degradation by human-associated Akkermansia
Source: J Bacteriol. 2024 Feb 1;206(2):e00334-23. doi: 10.1128/jb.00334-23 (PMC10886448; doi:10.1128/jb.00334-23)

Supplemental Figure 1

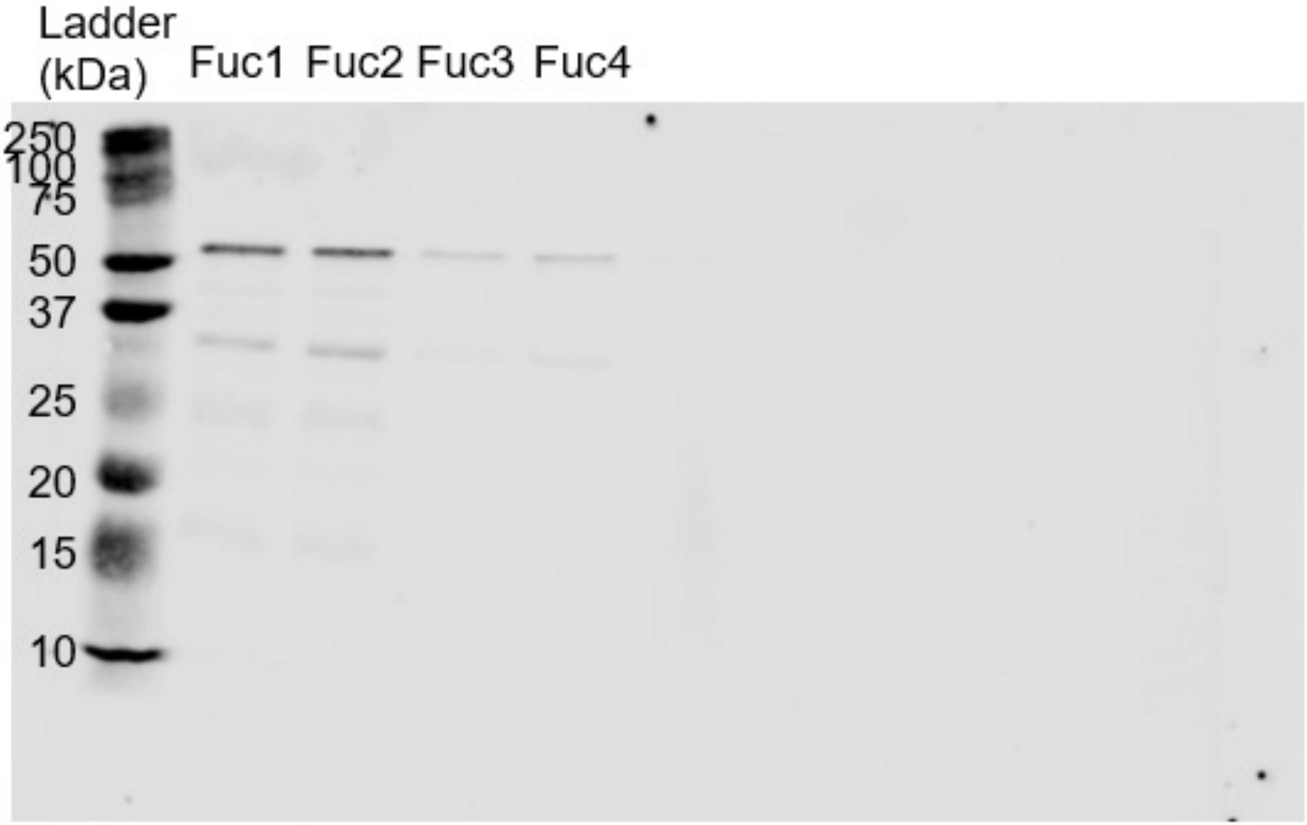

Supplemental Figure 2

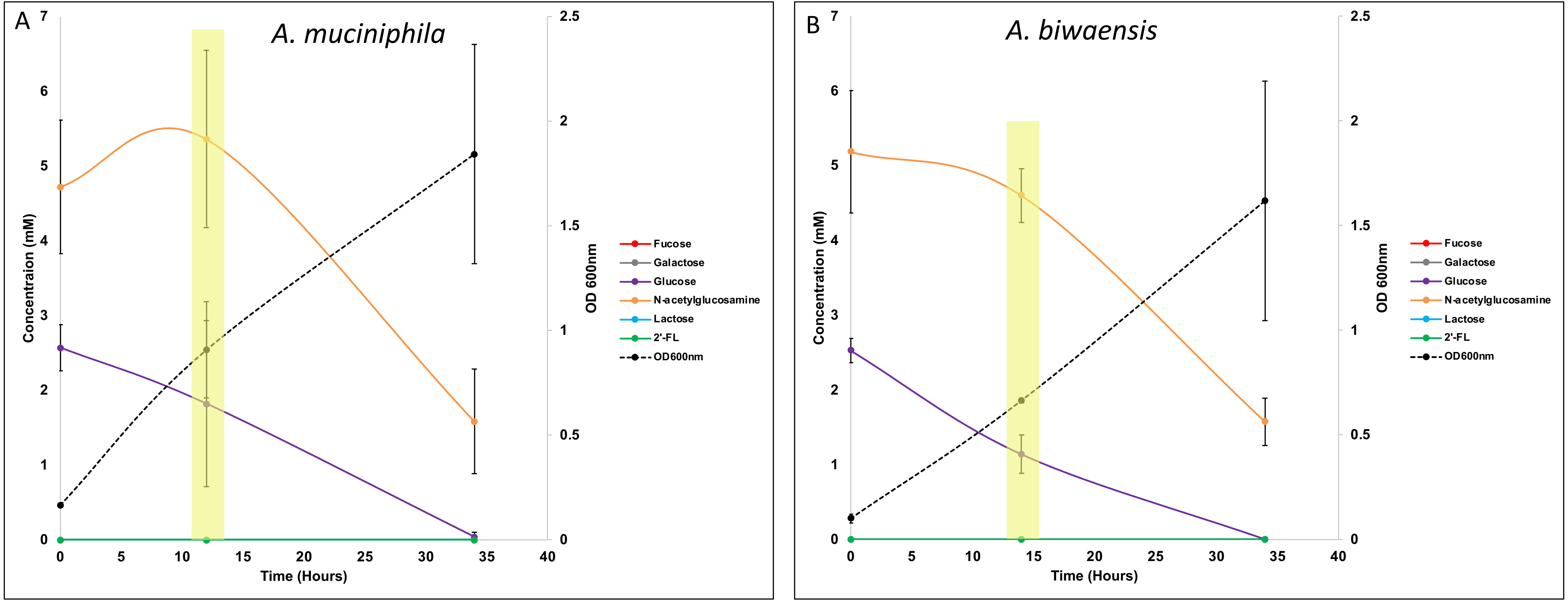

Supplemental Figure 3

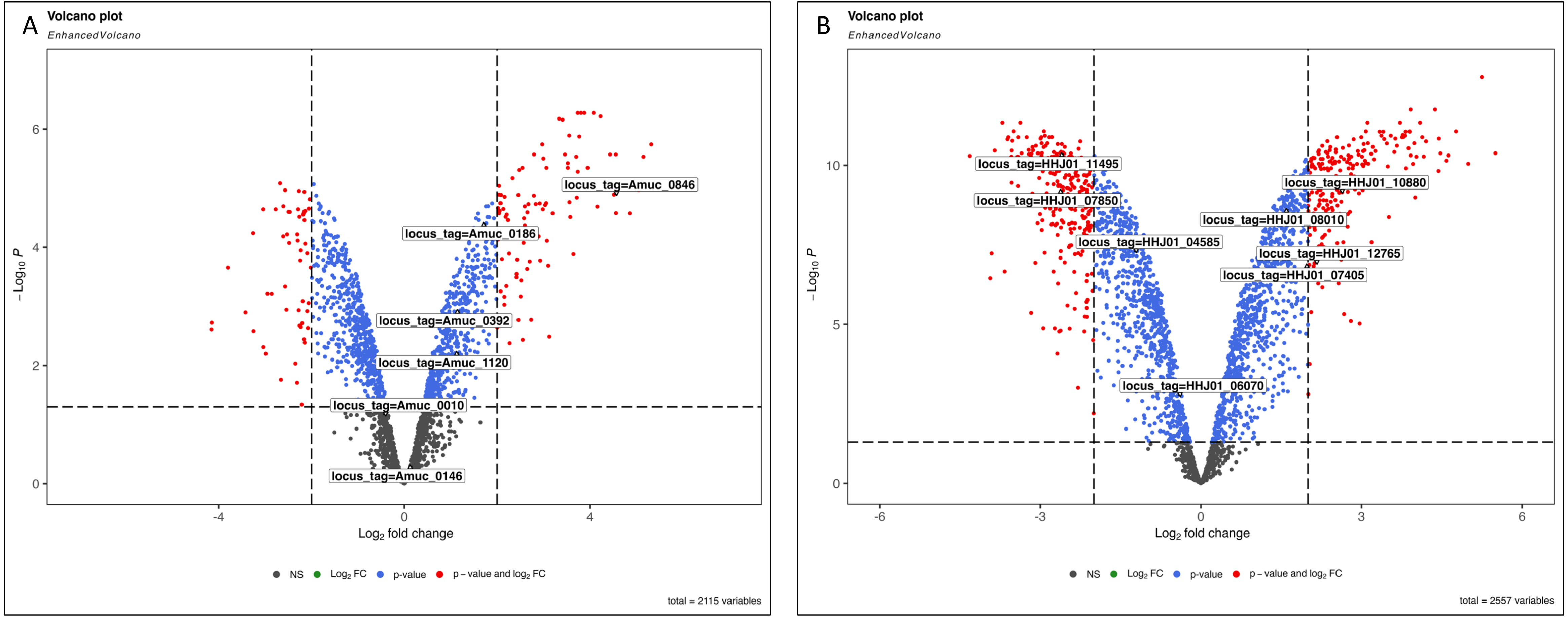

Supplemental Figure 4

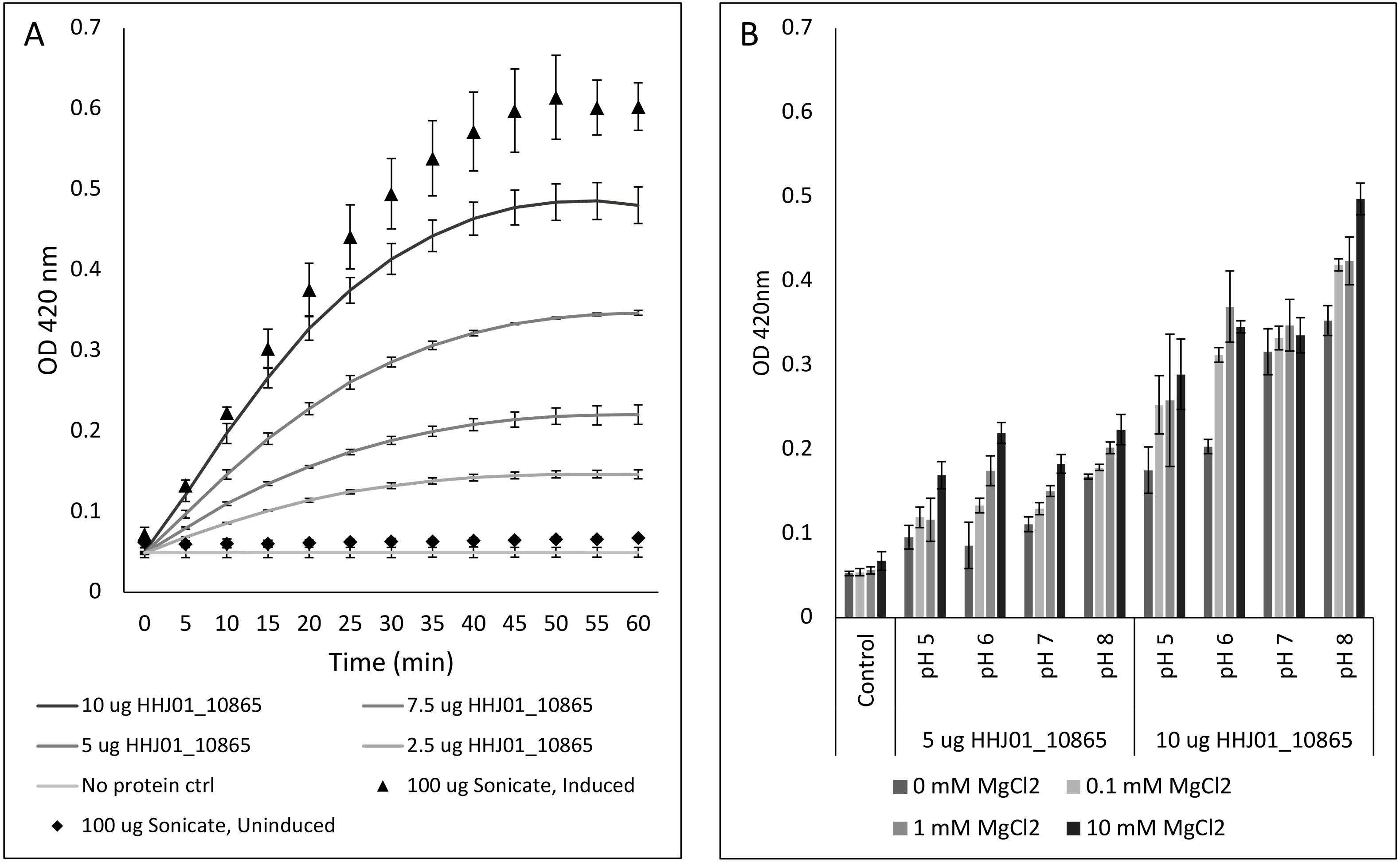

Supplemental Figure 5

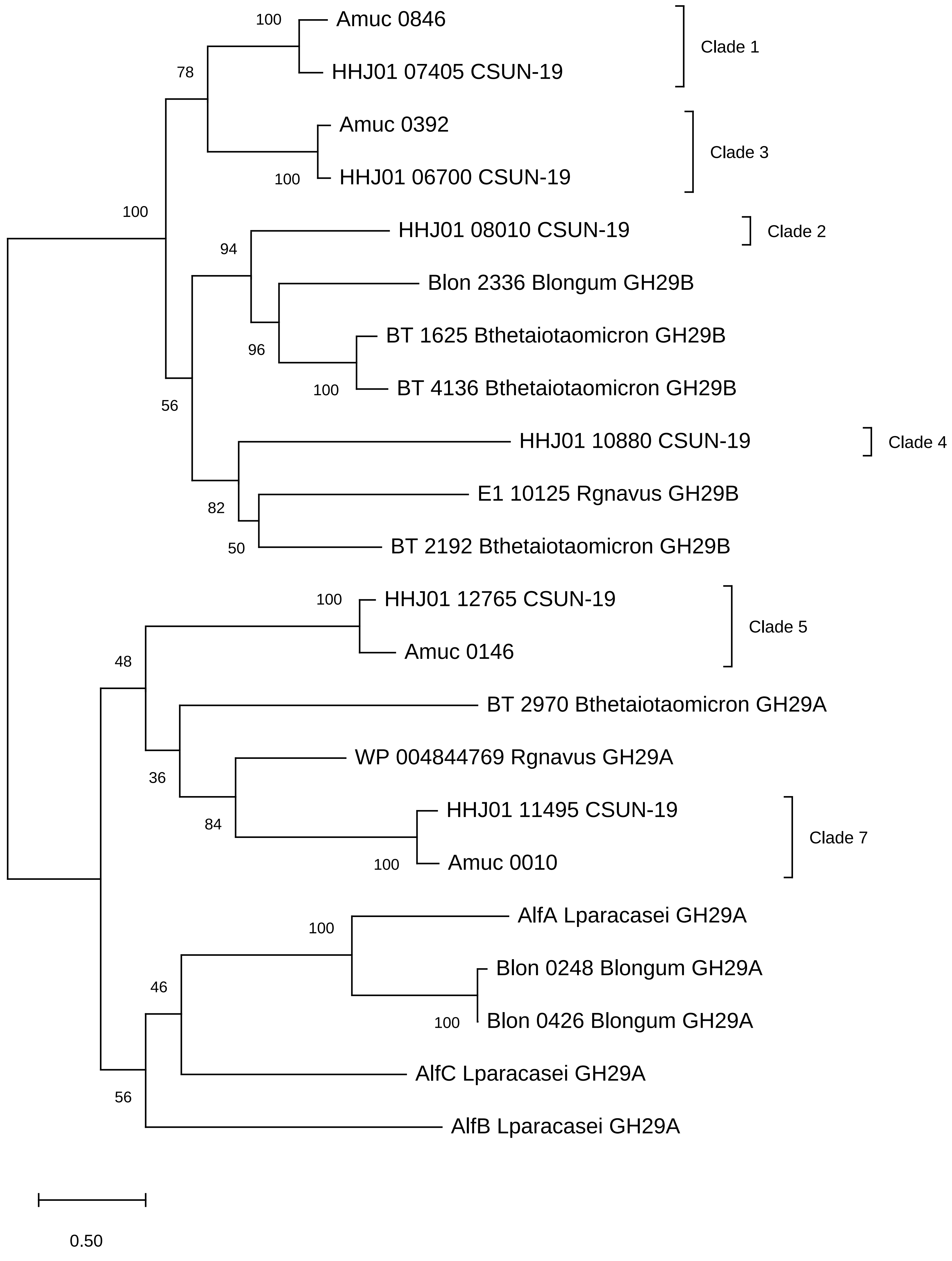

Supplemental Figure 6

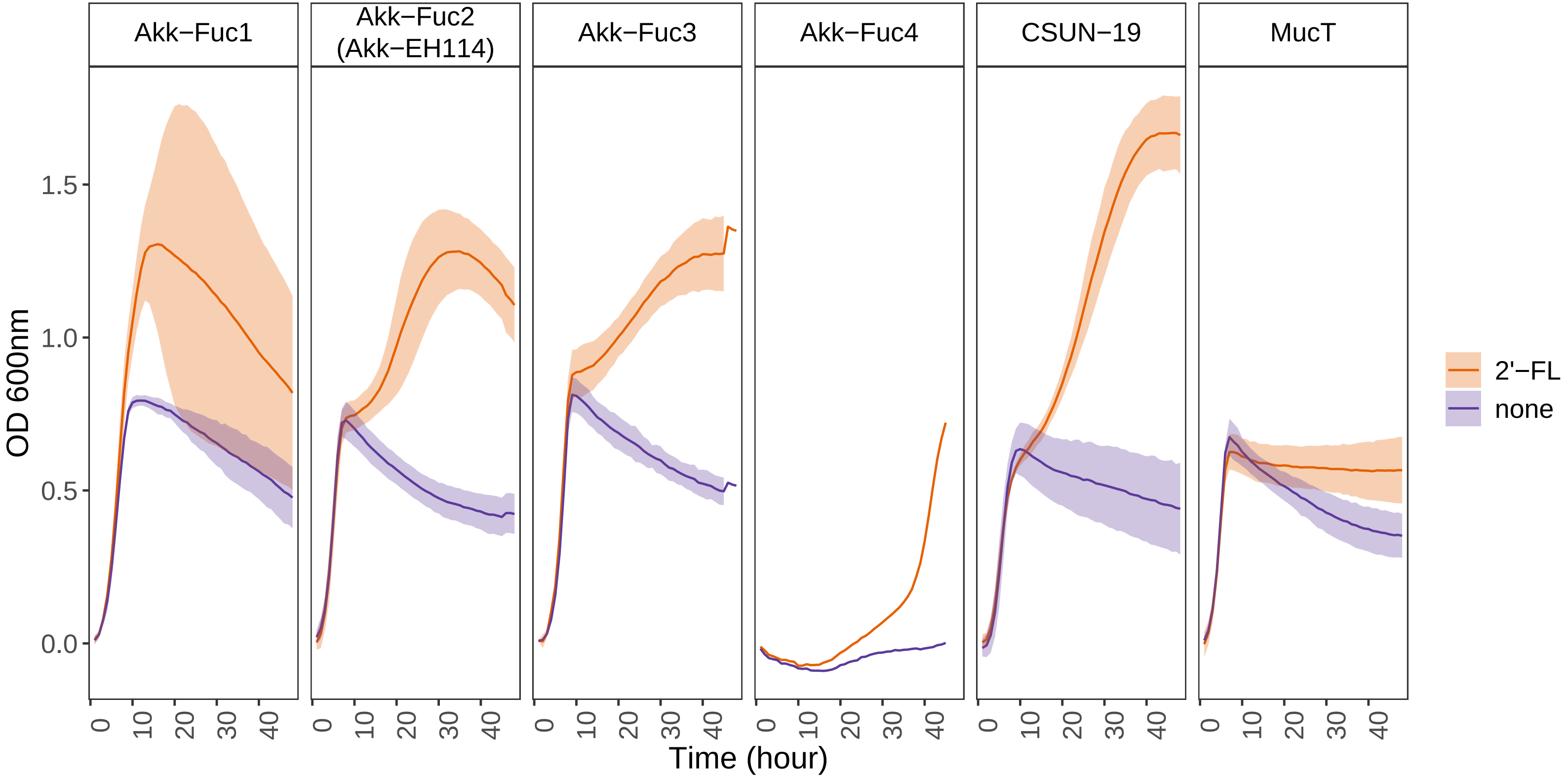

Supplement: Supplemental figures — Figures S1 to S6. [file jb.00334-23-s0003.pdf]
