# Supplementary material for: Mechanism of 2′-fucosyllactose degradation by human-associated Akkermansia
Source: J Bacteriol. 2024 Feb 1;206(2):e00334-23. doi: 10.1128/jb.00334-23 (PMC10886448; doi:10.1128/jb.00334-23)
Supplement: Tables S1 to S4 — Accession information for phylogenetic analyses and read information from RNAseq. [file jb.00334-23-s0005.pdf]

Supplemental Table 1

Strain	Accession/BioSample #	Phylogroup	GH2 copies	GH29 copies	GH95 copies	Reference
A. muciniphila Muc^T	CP001071	AmI	5	4	2	van Passel *et. al.*, 2011
A. muciniphila CSUN-7	SAMN14614183	AmI	6	4	2	Luna *et. al.*, 2022
A. muciniphila CSUN-12	SAMN14614184	AmI	6	4	2	Luna *et. al.*, 2022
A. massiliensis CSUN-17	SAMN14614185	AmII	6	5	3	Luna *et. al.*, 2022
A. biwaensis CSUN-19	SAMN14614186	AmIV	7	6	3	Luna *et. al.*, 2022
A. muciniphila CSUN-33	SAMN14614187	AmI	7	3	2	Luna *et. al.*, 2022
A. massiliensis CSUN-34	SAMN14614188	AmII	6	5	3	Luna *et. al.*, 2022
A. biwaensis CSUN-37	SAMN14614189	AmIV	7	6	3	Luna *et. al.*, 2022
A. massiliensis CSUN-50	SAMN14614190	AmII	6	5	3	Luna *et. al.*, 2022
Akkermansia sp. CSUN-56	SAMN14614191	AmIII	5	5	3	Luna *et. al.*, 2022
A. massiliensis CSUN-58	SAMN14614192	AmII	6	5	3	Luna *et. al.*, 2022
A. muciniphila CSUN-59	SAMN14614193	AmI	7	3	2	Luna *et. al.*, 2022
Akkermansia sp. GP22	SAMN08162556	AmIII	6	5	3	Guo *et. al.*, 2017
A. muciniphila Akk0096	SAMN18350232	AmI	6	4	2	Becken *et. al.*, 2021
A. muciniphila Akk0880	SAMN18350241	AmI	7	3	2	Becken *et. al.*, 2021
A. massiliensis Akk0580	SAMN18350240	AmII	6	5	3	Becken *et. al.*, 2021
A. biwaensis Akk0196	SAMN18350233	AmIV	7	6	3	Becken *et. al.*, 2021

Supplemental Table 2

Organism	Accession#	Locus Tag/ID	GH29 subfamily	Reference
**Bacteroides thetaiotaomicron VPI-5482**	AAO76732	BT_1625	GH29B	Shaikh et al., 2013
**Bacteroides thetaiotaomicron VPI-5482**	AAO78076	BT_2970	GH29A	Shaikh et al., 2013<br>Sakurama et al., 2012
**Bacteroides thetaiotaomicron VPI-5482**	AAO79241	BT_4136	GH29B	Shaikh et al., 2013
**Bacteroides thetaiotaomicron VPI-5482**	AAO77299	BT_2192	GH29B	Guillotin et al., 2014
**Bifidobacterium longum subsp. infantis ATCC 15697**	ACJ53394	Blon_2336	GH29B	Sela et al., 2012
**Bifidobacterium longum subsp. infantis ATCC 15697**	ACJ51376	Blon_0248	GH29A	Sela et al., 2012
**Bifidobacterium longum subsp. infantis ATCC 15697**	ACJ51546	Blon_0426	GH29A	Sela et al., 2012
***Lacticaseibacillus paracasei**	CAQ67115	AlfA	GH29A	Rodríguez-Díaz et al., 2011
***Lacticaseibacillus paracasei**	CAQ67877	AlfB	GH29A	Rodríguez-Díaz et al., 2011
***Lacticaseibacillus paracasei**	CAQ67984	AlfC	GH29A	Rodríguez-Díaz et al., 2011
**[Ruminococcus] gnavus E1**	6TR4_B	E1_10125	GH29B	Wu et al., 2021
**[Ruminococcus] gnavus ATCC 29149**	WP_004844769	-	GH29A	Wu et al., 2021

Supplemental Table 3

Primer Name	Description	Sequence (5’ -> 3’)
10865_F_Start_1	putative beta-galactosidase gene with **HindIII** and EcoR1 sites, partial signal sequence (20 aa removed)	TTT**AAGCTT**GAATTCATGTGCGGCCTGTCCGCCCATGGC
10865_F_Start_2	putative beta-galactosidase gene with **HindIII** and EcoR1 sites, no signal sequence (25 aa removed)	TTT**AAGCTT**GAATTCATGCATGGCGGGGATGCGCCTCCG
10865_R_1	putative beta-galactosidase gene HJ10865 with HindIII and **SalI** sites	**TGTCGACA**AGCTTTGGAGATTCCTGCTGTTTGTC

Strains	Genotype	Origin
NEB Turbo	K-12 glnV44 thi-1 Δ(lac-proAB) galE15 galK16 R(zgb-210::Tn10)TetS endA1 fhuA2 Δ(mcrB-hsdSM)5(rK–mK–) F’[traD36 proAB+ lacIq lacZΔM15]	Dr. Melissa Takahashi (California State University, Northridge, USA)
Tuner BL21	F– ompT hsdSB (rB– mB–) gal dcm lacY1(DE3)	Dr. Michael Summers (California State University, Northridge, USA)

Supplemental Table 4

Sample ID	Organism	Sugar	Concentration (ng/µL)	Total (ng)	RIN	Q30	# Reads	Pseudoaligned Reads
AmucT1	*A. muciniphila* Muc^T	Glc	122.00	2,440	5.8	95.49	15,354,070	5,715,392 (37%)
AmucT2	*A. muciniphila* Muc^T	Glc	41.40	828	5.6	95.46	16,183,428	6,313,357 (39%)
AmucT3	*A. muciniphila* Muc^T	Glc	125.00	2,500	5.8	95.54	15,657,002	5,763,870 (37%)
AmucT4	*A. muciniphila* Muc^T	2’-FL	9.50	190	9.8	95	15,044,641	5,211,036 (35%)
AmucT5	*A. muciniphila* Muc^T	2’-FL	10.20	204	9.6	95.12	13,766,275	4,312,559 (31%)
AmucT6	*A. muciniphila* Muc^T	2’-FL	0.92	18	9.4	92.29	15,896,122	4,396,982 (28%)
CSUN191	*A. biwaensis* CSUN-19	Glc	259.00	5,180	6.5	95.42	14,060,510	6,482,817 (46%)
CSUN192	*A. biwaensis* CSUN-19	Glc	273.00	5,460	8.3	95.48	16,576,896	7,786,699 (47%)
CSUN193	*A. biwaensis* CSUN-19	Glc	291.00	5,820	6.1	95.49	14,851,621	6,468,715 (44%)
CSUN194	*A. biwaensis* CSUN-19	2’-FL	101.00	2,020	6.7	95.01	14,166,132	4,951,006 (35%)
CSUN195	*A. biwaensis* CSUN-19	2’-FL	107.00	2,140	6.5	95.17	15,492,072	5,436,590 (35%)
CSUN196	*A. biwaensis* CSUN-19	2’-FL	78.00	1,560	6.2	95.06	15,231,567	5,985,852 (39%)
CSUN197	*A. biwaensis* CSUN-19	2’-FL	43.90	878	5.9	95.22	14,944,428	5,712,869 (38%)
