# Supplementary material for: Mechanism of 2′-fucosyllactose degradation by human-associated Akkermansia
Source: J Bacteriol. 2024 Feb 1;206(2):e00334-23. doi: 10.1128/jb.00334-23 (PMC10886448; doi:10.1128/jb.00334-23)
Supplement: Supplemental legends — Legends for all supplemental material. [file jb.00334-23-s0006.pdf]

SUPPLEMENTAL TABLE AND FIGURE LEGENDS

Supplemental Table 1. Table of Akkermansia strains used in this study. Accession numbers, phylogroup affiliation, and gene copy number for each strain is given.

Supplemental Table 2. Characterized glycosyl hydrolase family 29 (GH29) enzymes from other intestinal bacteria used in phylogenetic analysis with *A. muciniphila* Muc^{T} and *A. biwaensis* CSUN-19. Accession numbers, locus tag, GH29 sub-family, and reference for each sequence is given.

Supplemental File 1. R markdown file of basic code and outputs produced during analysis of *A. muciniphila* Muc^{T}.

Supplemental File 2. R markdown file of basic code and outputs produced during analysis of *A. biwaensis* CSUN-19.

Supplemental Table 3. Table of primers and strains used for cloning *E. coli* in this study. Primers used to clone HHJ01_10865 into pET28a for expression in the *E. coli* Tuner strain.

Supplemental Figure 1. Western blot of four *A. muciniphila* Muc^{T} strains expressing HA-tagged HHJ01_10880 from *A. biwaensis*. A transposon (Tn) encoding HA-tagged HHJ01_10880 was delivered into *A. muciniphila* Muc^{T} by conjugation and four Cm^{R} colonies were characterized by western blot with anti-HA antibodies. Tn insertion sites were identified by inverse PCR and found to occur at *Amuc_1192* (Fuc 1), *Amuc_2072* (Fuc 2), *Amuc_1984* (Fuc 3), and *Amuc_0172* (Fuc 4). Clone #2 (Fuc2) was renamed Akk-EHY114 and used in functional assays.

Supplemental Figure 2. *Akkermansia* species *A. muciniphila* Muc^{T} and *A. biwaensis* CSUN-19 exhibit similar growth kinetics on glucose (Glc) and N-acetylglucosamine (GlcNAc). *A. muciniphila* Muc^{T} (**A**) and *Akkermansia* species CSUN-19 (**B**) degrade Glc (purple line) concomitantly with GlcNAc (orange line) during growth as measured by OD_{600nm} (black dashed line). Areas highlighted in yellow denote time points where cells were collected for RNA extraction and RNAseq. Error bars represent standard deviation of triplicate cultures.

Supplemental Table 4. Whole genome transcriptional profiling resulted in high quality reads matching to the respective genomes.
Final measured RNA concentration for each extraction varied within and across samples. Reads were aligned with Kallisto resulting in approximately thirty eight percent non-ribosomal matching to the corresponding coding sequences from each genome.

Supplemental Figure 3. Glycosyl hydrolases genes are regulated during growth in synthetic medium with N-acetylglucosamine (GlcNAc) and 2'-fucosyllactose (2'-FL). Volcano plot of differentially expressed genes of *A. muciniphila* Muc^{T} (**A**) and *A. biwaensis* CSUN-19 (**B**) when grown in synthetic medium with 2'-FL versus glucose. Each dot represents a gene (grey), and colors indicate a P value <0.05 (blue) with a Log2 fold change $\geq$ 2 (red). Putative fucosidase genes are labeled for *A. muciniphila* (GH29: Amuc_0846, Amuc_0392, Amuc_0010; GH95: Amuc_0186, Amuc_1120) and *A.*

biwaensis CSUN-19 (GH29: HHJ01_10880, HHJ01_11495, HHJ01_12765, HHJ01_08010, HHJ01_07405, HHJ01_06700; GH95: HHJ01_07850, HHJ01_04585, HHJ01_06070).

Supplemental Table 5. Full table of differentially expressed genes for *A. muciniphila* Muc^T on 2’-FL vs Glucose. For each locus tag, fold change, average expression, and adjusted p-value generated by *limma* in R. The annotations were generated by the NCBI Prokaryotic Genome Annotation Pipeline (PGAP).

Supplemental Table 6. Full table of differentially expressed genes for *A. biwaensis* CSUN-19 on 2’-FL vs Glucose. For each locus tag, fold change, average expression, and adjusted p-value generated by *limma* in R. The annotations were generated by the NCBI Prokaryotic Genome Annotation Pipeline (PGAP).

Supplemental Figure 4. Recombinant GH2 protein from *A. biwaensis* CSUN-19, HHJ01_10865, has β-galactosidase activity that is strongest at pH 8 with 10 mM $MgCl_2$. Different concentrations of purified HHJ01_10865 protein or cell lysates from uninduced and induced fractions were incubated at 37ºC and pH8 with 10 mM $MgCl_2$ and 2.5 mM ONPG and read every five minutes over the course of an hour at OD_{420nm} (**A**). Purified protein was incubated at 37ºC at a range of pH and $MgCl_2$ concentrations with ONPG and read at OD_{420nm} after one hour (**B**). Error bars represent standard deviation of three technical replicates.

Supplemental Figure 5: Phylogenetic analysis comparing the amino acid sequences of GH29 genes from *A. muciniphila* Muc^T and *A. biwanesis* CSUN-19 with characterized enzymes from other gut bacteria. Note how HHJ01_10880 clusters with GH29B enzymes from *Bacteroides thetaiotaomicron* and *Ruminococcus gnavus*, both known mucin and HMO degrading bacteria. Non-*Akkermansia* genes are referenced in Supplemental Table X, while clade designations follow those defined in Figure 1. The evolutionary history was inferred by using the Maximum Likelihood method and JTT matrix-based model. The tree with the highest log likelihood (-23546.66) is shown. The percentage of trees (n=50) in which the associated taxa clustered together is shown next to the branches. Initial tree(s) for the heuristic search were obtained automatically by applying Neighbor-Join and BioNJ algorithms to a matrix of pairwise distances estimated using the JTT model, and then selecting the topology with superior log likelihood value. The tree is drawn to scale, with branch lengths measured in the number of substitutions per site. This analysis involved 22 amino acid sequences. There were a total of 1,065 positions in the final dataset. Evolutionary analyses were conducted in MEGA11 (44–46).

Supplemental Figure 6. All four clones of *A. muciniphila*, including strain Akk-EH114, containing the GH29 from *A. biwaensis* CSUN-19 (HHJ01_10880) display increased growth on 2’-FL. Cultures were inoculated into BTTM containing a final concentration of 0.4% Mucin and 10 mM 2’-FL. Optical density (OD_{600nm}) was read every hour for 48h and data was normalized to time zero. Due to poor growth of the fourth clone, this construct was only grown once, otherwise ribbons represent standard deviation of two to three biological replicates.
